# ﻿Two new species of *Ismarus* Haliday (Hymenoptera, Ismaridae) from Yunnan, China

**DOI:** 10.3897/zookeys.1174.106404

**Published:** 2023-08-10

**Authors:** Cheng-Jin Yan, Yan-Qiong Peng, Hua-Yan Chen

**Affiliations:** 1 Southern Zhejiang Key Laboratory of Crop Breeding, Wenzhou Vocational College of Science and Technology, Wenzhou, 325006, China Southern Zhejiang Key Laboratory of Crop Breeding, Wenzhou Vocational College of Science and Technology Wenzhou China; 2 CAS Key Laboratory of Tropical Forest Ecology, Xishuangbanna Tropical Botanical Garden, Chinese Academy of Sciences, Mengla 666303, China CAS Key Laboratory of Tropical Forest Ecology, Xishuangbanna Tropical Botanical Garden, Chi-nese Academy of Sciences Mengla China; 3 Key Laboratory of Plant Resources Conservation and Sustainable Utilization, South China Botanical Garden, Chinese Academy of Sciences, Guangzhou 510650, China Key Laboratory of Plant Resources Conservation and Sustainable Utilization, South China Botanical Garden, Chinese Academy of Sciences Guangzhou China

**Keywords:** Diaprioidea, hyperparasitoid, key, new species, taxonomy, wasp

## Abstract

The genus *Ismarus* Haliday are rarely collected parasitoids in the small family Ismaridae. In this study, two new species are described from China’s Yunnan Province: *Ismarusrobustus* Chen & Yan, **sp. nov.** and *Ismarusunisulcus* Chen & Yan, **sp. nov.** An updated key to the Chinese species of the genus is provided.

## ﻿Introduction

*Ismarus* Haliday belongs to the small parasitoid wasp family Ismaridae, with 59 described species worldwide (Masner 1976; Johnson 1992; Liu et al. 2011; Comério et al. 2016; Kolyada and Chemyreva 2016; Kim et al. 2018a, b; Zhang et al. 2021). Previous studies found that species of *Ismarus* are hyperparasitoids of Dryinidae, which are primary parasitoids of leafhoppers, planthoppers and treehoppers (Chambers 1955, 1981; Nixon 1957; Wall 1967; Kozlov 1971; Masner 1976; Jervis 1979; Tussac and Tussac 1991; Olmi 2000). Studies also suggested that species of *Ismarus* prefer wooded areas at higher elevations in warmer climatic zones and at low elevations in cooler climatic zones (Masner 1976; Kim et al. 2018a, b), although some species have been found to occur in warm subtropical regions (Zhang et al. 2021).

The Chinese fauna of *Ismarus* has been extensively studied recently and nine species (Table 1) have been recorded (Liu et al. 2011; Kim et al. 2018b; Zhang et al. 2021). In this study, we describe another two new species from the mountainous region of Yunnan Province of southwest China.

**Table 1.** An updated list of the Chinese species of *Ismarus* with their distribution in China.

Species	Distribution in China	
	Province	Realm
*Ismarusapicalis* Kolyada & Chemyreva	Jilin	Palearctic
*Ismarusareolatus* Chen	Guangdong	Indomalayan
*Ismarusexcavatus* Kim & Lee	Jilin	Palearctic
*Ismarushalidayi* Foerster	Sichuan, Guizhou, Yunnan, Tibet	Indomalayan
	Ningxia	Palearctic
*Ismaruslongus* Liu, Chen & Xu	Yunnan	Indomalayan
*Ismarusnigritrochanter* Liu, Chen & Xu	Yunnan	Indomalayan
*Ismarusparadorsiger* Chen	Guangdong	Indomalayan
*Ismarusparvicellus* Liu, Chen & Xu	Hainan	Indomalayan
*Ismarusspinalis* Kolyada & Chemyreva	Heilongjiang	Palearctic
*Ismarusrobustus* Chen & Yan, sp. nov.	Yunnan	Indomalayan
*Ismarusunisulcus* Chen & Yan, sp. nov.	Yunnan	Indomalayan

## ﻿Material and method

This work is based on the specimens collected by Malaise trap set in Gaoligongshan National Nature Reserve, Yunnan Province, China. All the studied specimens are deposited in the insect collection of the South China Botanical Garden, Chinese Academy of Sciences, Guangzhou, China (SCBG).

Abbreviations and morphological terms used in the text: **A1, A2, ... A12**: antennomere 1, 2, … 12; **POL**: posterior ocellar line (shortest distance between posterior ocelli); **OOL**: ocular ocellar line (shortest distance between posterior ocellus and compound eye); **T1, T2, ... T8**: metasomal tergite 1, 2, ... 8. Morphological terminology otherwise follows Masner (1976) and Zhang et al. (2021). Species of *Ismarus* from China have been thoroughly reviewed and keyed (Zhang et al. 2021), which provides us a useful tool to identify the species in this study.

Specimens were examined using a Nikon SMZ800N microscope. Images and measurements were made using a Nikon SMZ25 microscope with a Nikon DS-Ri 2 digital camera system. Image plates were post-processed with Adobe Photoshop CS6 Extended.

## ﻿Taxonomy

### ﻿ Ismarus

Taxon classificationAnimaliaHymenopteraIsmaridae

Haliday, 1835

1F2FD612-8094-5ACF-8339-EB243C1FA30A

Ismarus Haliday, 1835: 467. Type species Cinetusdorsiger Haliday, 1831, by monotypy.
Entomia Herrich-Schäffer, 1840: 127. Type species Entomiacampanulata Herrich-Schäffer, 1840, by monotypy.
Agonophorus Dahlbom, 1858: 289. Type species Ismarusrugulosus Förster, 1850, designated by Muesebeck (1972).

#### Diagnosis.

Low insertion of antennae; antennal shelf not developed; antenna of female 15-merous and male 14-merous; notauli absent; mesoscutual suprahumeral sulcus absent or present as a single pit or several pits; mesoscutum strongly arched in lateral view; base of second tergite with median furrow (Masner 1976; Zhang et al. 2021).

### ﻿Key to species of *Ismarus* from China (females)

**Table:** 

1	Body mostly pale to bright yellow (fig. 12A in Zhang et al. 2021); suture separating T2 and T3 incomplete (fig. 12F in Zhang et al. 2021)	***Ismarusparadorsiger* Chen**
–	Body mostly black or dark brown (Figs 1A, 3A); suture separating T2 and T3 complete (Figs 1F, 3F)	**2**
2	Posterior surface of mesoscutellum areolate (fig. 2C in Zhang et al. 2021); lateral pronotal area with an oblique submedian carina (fig. 2D in Zhang et al. 2021)	***Ismarusareolatus* Chen**
–	Posterior surface of mesoscutellum smooth (Figs 1C, 3C); lateral pronotal area without carina (Figs 1D, 3D)	**3**
3	Radial cell of fore wing distinctly shorter than marginal vein (figs 8A, 10A, 16A in Zhang et al. 2021)	**4**
–	Radial cell of fore wing slightly shorter or as long as marginal vein (Figs 2A, 4A)	**6**
4	Mesoscutal suprahumeral sulcus present as a single pit that is longer than length of the mesoscutellar disc (fig. 15C in Zhang et al. 2021); radial cell of fore wing 0.3× length of marginal vein (fig. 16A in Zhang et al. 2021)	***Ismarusparvicellus* Liu, Chen & Xu**
–	Mesoscutal suprahumeral sulcus present as four or five foveae of varying size (figs 7C, 9C in Zhang et al. 2021); anterior mesoscutellar pit distinctly shorter than length of the mesoscutellar disc (figs 7C, 9C in Zhang et al. 2021); radial cell of fore wing 0.6× length of marginal vein (figs 8A, 10A in Zhang et al. 2021)	**5**
5	Second flagellomere 5.0× as long as wide (fig. 8B in Zhang et al. 2021); radial cell of fore wing 3.0× as long as high (fig. 8A in Zhang et al. 2021); antenna black with scape brown, pedicel and first flagellomere dark brown (fig. 8B in Zhang et al. 2021); all trochanters brown (fig. 7A in Zhang et al. 2021)	***Ismaruslongus* Liu, Chen & Xu**
–	Second flagellomere 3.5× as long as wide (fig. 10B in Zhang et al. 2021); radial cell of fore wing 2.0× as long as high (fig. 10A in Zhang et al. 2021); antenna uniformly black (fig. 10B in Zhang et al. 2021); all trochanters black (fig. 9A in Zhang et al. 2021)	***Ismarusnigritrochanter* Liu, Chen & Xu**
6	Antenna uniformly bright yellow or only A15 brown (figs 22, 23 in Kolyada and Chemyreva 2016)	**7**
–	Antenna not bright yellow, variable (Figs 2B, 4B)	**8**
7	Antenna uniformly bright yellow, including A15 (fig. 22 in Kolyada and Chemyreva 2016); anterior mesoscutellar pit with median keel; radial cell of fore wing as long as marginal vein (fig. 17 in Kolyada and Chemyreva 2016)	***Ismarusspinalis* Kolyada & Chemyreva**
–	Antenna bright yellow, except A15 brown (fig. 23 Kolyada and Chemyreva 2016); anterior mesoscutellar pit without median keel; radial cell of fore wing 0.8× length of marginal vein (fig. 12 Kolyada and Chemyreva 2016)	***Ismarusapicalis* Kolyada & Chemyreva**
8	Mesoscutal suprahumeral sulcus present as six small pits (Fig. 1C); antenna entirely black (Fig. 2B)	***Ismarusrobustus* Chen & Yan, sp. nov.**
–	Mesoscutal suprahumeral sulcus present as a single anterior pit (Fig. 3C); antenna brown to dark brown (Fig. 4B)	**9**
9	Median furrow of T2 long, half the length of T2 (fig. 4E in Zhang et al. 2021)	***Ismarushalidayi* Förster**
–	Median furrow of T2 very short, distinctly less than half the length of T2 (Fig. 3E)	**10**
10	Base of T2 with several short costae forming several furrows; A10–A15 dark brown (fig. 5A in Kim et al. 2018b)	***Ismarusexcavatus* Kim & Lee**
–	Base of T2 with two costae forming a single furrow (Fig. 3E); A10–A15 black (Fig. 4B)	***Ismarusunisulcus* Chen & Yan, sp. nov.**

### ﻿ Ismarus robustus

Taxon classificationAnimaliaHymenopteraIsmaridae

Chen & Yan sp. nov.

E448B6F8-14BA-57DE-82FB-66D3CBD319E8

https://zoobank.org/CC12F866-7B9F-4DB3-8426-AA29B70309A9

Figs 1 , 2

#### Material examined.

***Holotype*. China**•1♀; Yunnan, Gaoligongshan National Nature Reserve, Dulong River, GLG12; 27°53'51.96"N, 98°20'11.89"E, 1496 m; May–Jun. 2020; Lang Yi leg.; Malaise trap; SCBG 3044338.

**Figure 1.**
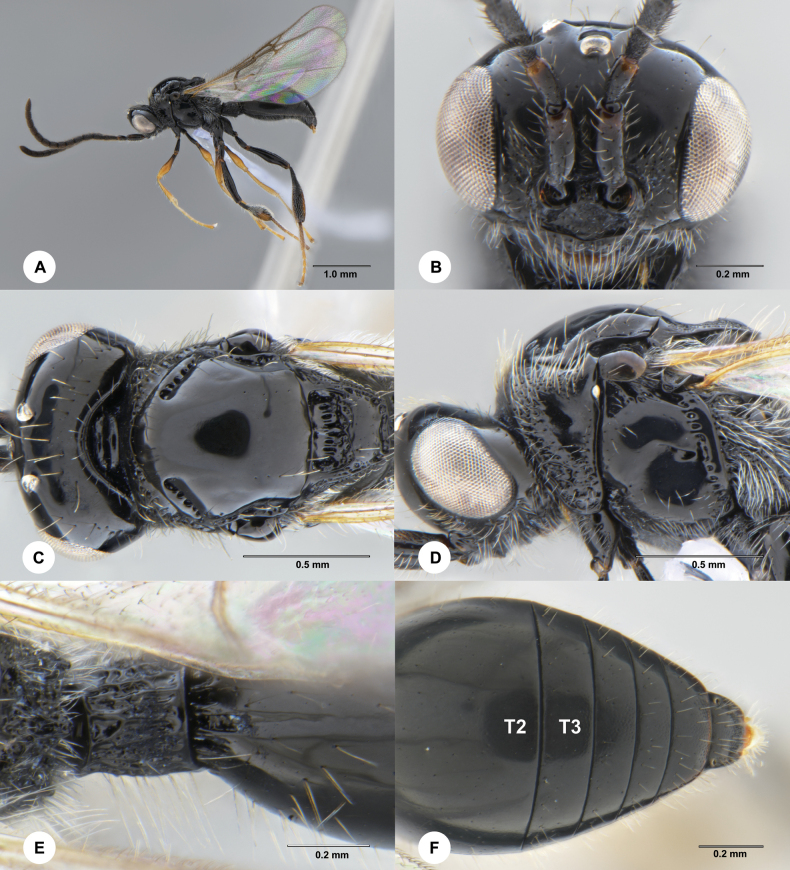
*Ismarusrobustus* Chen & Yan, sp. nov., holotype, female (SCBG 3044338) **A** lateral habitus **B** head, anterior view **C** head and mesosoma, dorsal view **D** head and mesosoma, lateral view **E** apex of propodeum and basal metasoma, dorsal view **F** posterior half of T2 and T3 to T7, dorsal view.

#### Diagnosis.

This species can be easily distinguished from other *Ismarus* species by the following characters: largely black; mesoscutal suprahumeral sulcus present as six small pits; mesoscutellum with posterior rim excavate and slightly prominent posterolateral corners; hind tibia abruptly incrassate.

**Figure 2.**
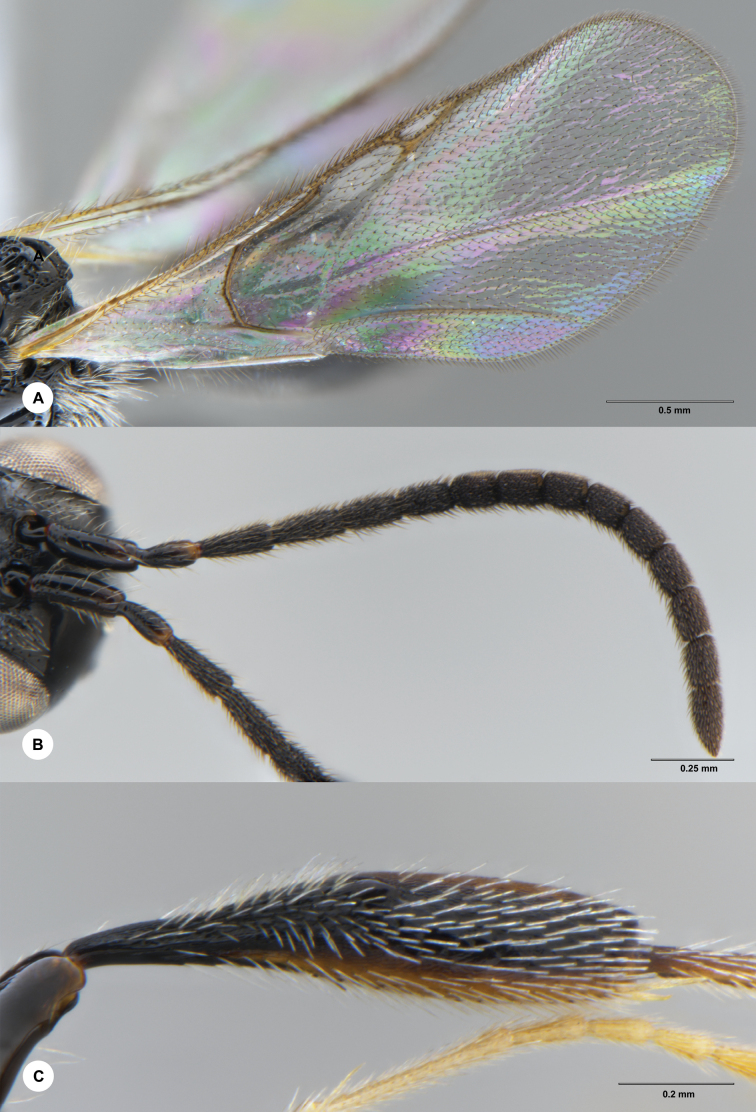
*Ismarusrobustus* Chen & Yan, sp. nov., holotype, female (SCBG 3044338) **A** fore wing **B** antenna **C** hind tibia.

#### Description.

**Female.** Body length 3.33 mm.

***Colour*.** Body black; antenna entirely black; coxae, trochanters and basal femora of fore and mid legs dark, remainder brown to yellow, with tibia and tarsi becoming paler distally, hind leg mostly black with hind tibia laterally yellow-brown and hind tarsi pale yellow; wings hyaline, veins brown to black-brown.

***Head*.** Head 2.0× as wide as long in dorsal view; vertex abruptly sloping behind ocelli in lateral view; POL as long as OOL; most of frons with scattered setae, except densely setose ventro-laterally; transverse facial carina convex ventrally; A3 as long as A4; A4 1.3× length of A5; A6–A14 with each segment approximately 1.4× longer than wide; A15 approximately 2.5× longer than wide.

***Mesosoma*.** Dorsal pronotal area punctate and setose; lateral pronotal area rugose-punctate ventrally, smooth dorsally; mesoscutum smooth, shiny and convex; mesoscutal suprahumeral sulcus present as six small pits; mesoscutal humeral sulcus deep and finely crenulate, 1.6× length of tegula; mesoscutellum convex with scattered punctae, posterior rim excavate with slightly prominent posterolateral corners; anterior mesoscutellar pit large and deep, as long as the length of the mesoscutellar disc, distinctly crenulate medially, rugose-punctate posteriorly, median keel strong; mesopleuron smooth, with area below tegula rugulose; metapleuron rugose-punctate and covered with dense, long whitish setae.

***Wings*.** Radial cell completely closed, moderately large, 5.0× as long as wide and 0.7× as long as marginal vein.

***Legs*.** Fore and mid legs slender; hind tibia abruptly incrassate, its maximum width slightly wider than hind femur.

***Metasoma*.** Petiole slightly shorter than wide (8:9), with irregular longitudinal costae dorsally; tergites smooth with scattered fine punctures; base of T2 with several short costae and short median furrow, extending 0.27× length of T2; sutures between tergites complete and deeply impressed.

**Male.** Unknown.

#### Etymology.

Named after the comparatively robust body of this species.

#### Distribution.

China (Yunnan).

### ﻿ Ismarus unisulcus

Taxon classificationAnimaliaHymenopteraIsmaridae

Chen & Yan sp. nov.

529E0C8A-7098-5A41-B50F-7A305447855B

https://zoobank.org/25B2BB9E-00E3-44AC-B9BB-CB6B5A7F1D60

Figs 3 , 4

#### Material examined.

***Holotype*. China**•1♀; Yunnan Province, Gaoligongshan National Nature Reserve, Dulong River, GLG13; 27°50'55.81"N, 98°28'3.15"E, 2824 m; 2–15 Jul. 2020; Lang Yi leg.; Malaise trap; SCBG 3044337. ***Paratype*. China**•1♀; same locality as holotype, but 2–16 Jul. 2020; Lang Yi leg.; Malaise trap; SCBG 3049369.

**Figure 3.**
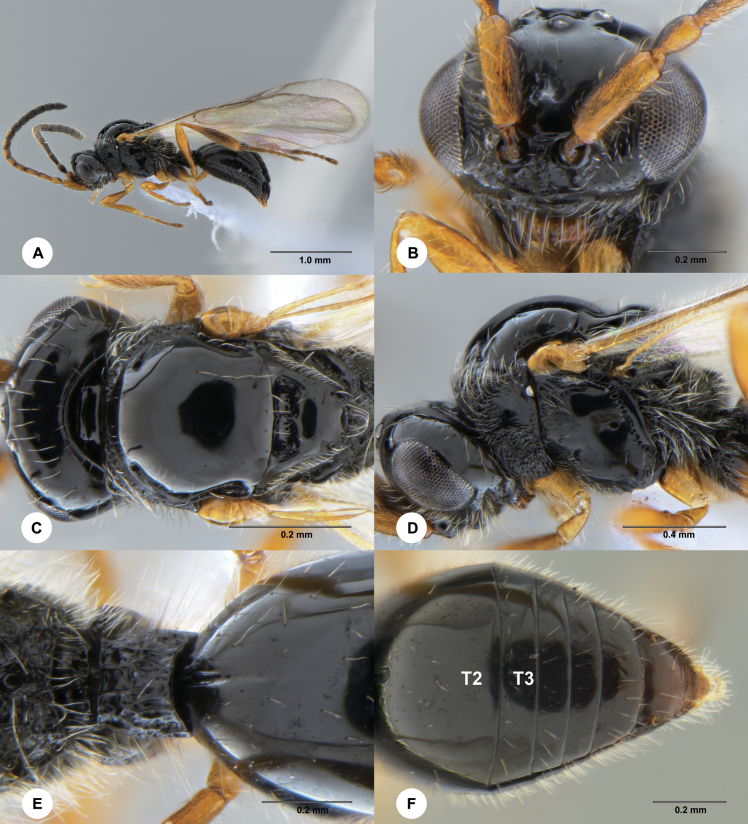
*Ismarusunisulcus* Chen & Yan, sp. nov., holotype, female (SCBG 3044337) **A** lateral habitus **B** head, anterior view **C** head and mesosoma, dorsal view **D** head and mesosoma, lateral view **E** apex of propodeum and basal metasoma, dorsal view **F** apical metasoma, dorsal view.

#### Diagnosis.

This species is most similar to *I.halidayi* Förster but can be distinguished by the following characters: A4 slightly longer than A3 (A4 shorter than A3 in *I.halidayi*); median furrow of T2 very short, distinctly less than half the length of T2 (median furrow of T2 long, reaching half the length of T2 in *I.halidayi*); radial cell of fore wing 0.75× as long as marginal vein (radial cell as long as marginal vein in *I.halidayi*).

**Figure 4.**
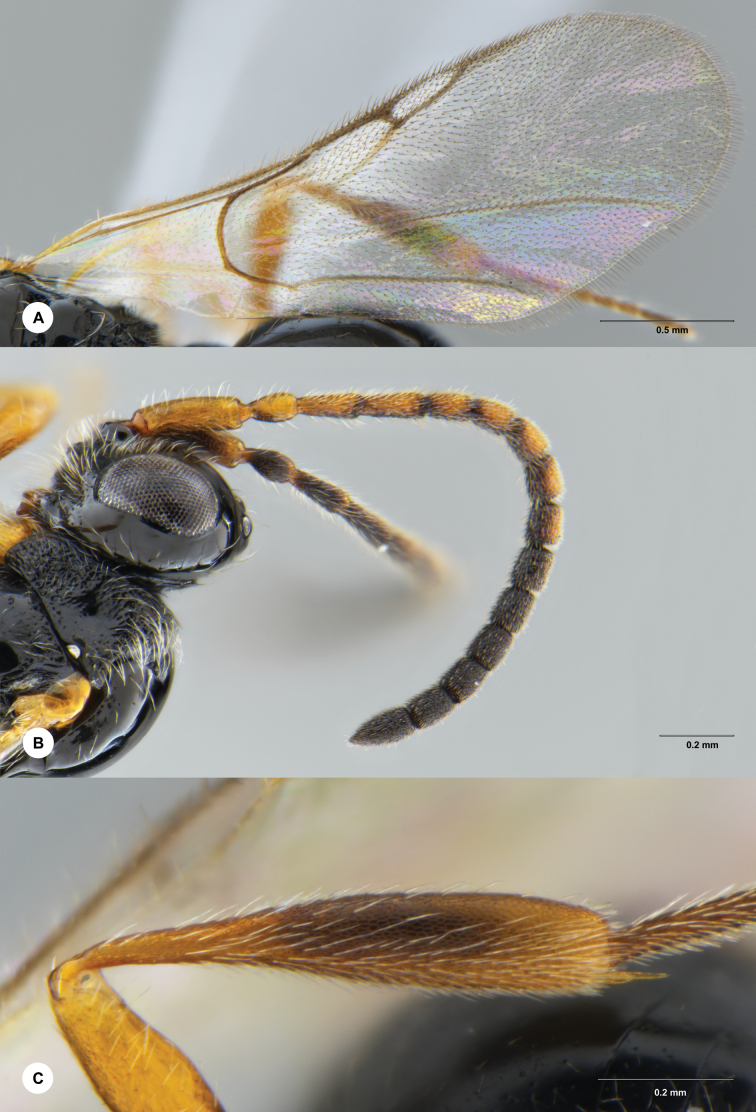
*Ismarusunisulcus* Chen & Yan, sp. nov., holotype, female (SCBG 3044337) **A** fore wing **B** antenna **C** hind tibia.

#### Description.

**Female.** Body length 2.50–2.64 mm.

***Colour*.** Body black; A1–A9 brown to dark brown, remainder of antenna dark brown; fore and mid legs yellow-brown, with tarsi becoming darker distally, hind leg mostly dark brown with basal coxae somewhat dark and hind femur, trochanter and basal tibia yellow-brown; tegulae yellow-brown; wings hyaline, veins light brown to black-brown.

***Head*.** Head 2.0× as wide as long in dorsal view; vertex abruptly sloping behind ocelli in lateral view; POL as long as OOL; most of frons with scattered setae, except densely setose ventro-laterally; transverse facial carina slightly convex ventrally; A4 slightly longer than A3; A4 1.5× length of A5; A6–A14 with each segment less than 1.5× as long as wide; A15 approximately 2.0× longer than wide.

***Mesosoma*.** Dorsal pronotal area rugose-punctate and setose; lateral pronotal area rugose-punctate ventrally, smooth dorsally; mesoscutum smooth, shiny and convex, posterior margin with scattered long setae; mesoscutal suprahumeral sulcus present as a single anterior pit; mesoscutal humeral sulcus as long as tegula, deep and crenulate; mesoscutellum smooth and slightly convex, posterior rim rounded; anterior mesoscutellar pit large and deep, shorter than length of the mesoscutellar disc, sparsely punctate posteriorly, median keel weakly defined; mesopleuron smooth, with area below tegula rugulose; metapleuron rugose-punctate and covered with dense, long whitish setae.

***Wings*.** Radial cell closed, moderately large, 5.6× as long as wide and 0.94× as long as marginal vein.

***Legs*.** Fore and mid legs slender; hind tibia gradually swollen, its maximum width slightly wider than hind femur.

***Metasoma*.** Petiole slightly shorter than wide (8:9), with strong costae dorsally; tergites smooth with scattered fine punctures; base of T2 with two short costae and a short median furrow, extending 0.37× length of T2; sutures between tergites complete and deeply impressed.

**Male.** Unknown.

#### Etymology.

The name refers to the single furrow present on the base of T2.

#### Distribution.

China (Yunnan).

## Supplementary Material

XML Treatment for Ismarus

XML Treatment for Ismarus robustus

XML Treatment for Ismarus unisulcus

## ﻿Additional information

### Author ORCIDs

Cheng-Jin Yan https://orcid.org/0000-0001-5723-9635

Yan-Qiong Peng https://orcid.org/0000-0002-7453-9119

Hua-Yan Chen https://orcid.org/0000-0002-0382-1635

### Data availability

All of the data that support the findings of this study are available in the main text.
